# Living Lab-Based Service Interaction Design for a Companion Robot for Seniors in South Korea

**DOI:** 10.3390/biomimetics8080609

**Published:** 2023-12-14

**Authors:** Ju Yeong Kwon, Da Young Ju

**Affiliations:** Department of AI Design & Design Science, Kookmin University, 77 Jeongneung-ro, Seongbuk-gu, Seoul 02707, Republic of Korea; juyeong@kookmin.ac.kr

**Keywords:** living lab, digital companion robot, service interaction design, seniors

## Abstract

A living lab is a valuable method for designing tangible and intangible service elements, ensuring a comprehensive user experience. Developing a digital companion service, which users may be unfamiliar with, requires observing user behavior in real-world environments and analyzing living and behavioral patterns. A living lab starts with understanding user characteristics and behaviors. Living lab methods have an impact on the accuracy and precision of service design. The number of seniors in South Korea is rapidly increasing, leading to a rise in social issues like solitary deaths and suicide. Addressing these problems has led to a growing demand for companion robots. To design effective companion services, understanding seniors’ living environments and their cognitive and behavioral traits is essential. This opinion piece, based on a national R&D project, presents the development of a digital companion for seniors. It offers insights, providing a comprehensive overview of living lab-based service interaction design and proposing methodologies about living lab environment construction and experimentation and considerations when designing robot interaction functions and appearance. The living lab environment includes real living spaces, laboratories, virtual reality settings, and senior welfare centers. Using the research findings, we created service scenarios, analyzed senior language characteristics, and developed the concept and facial expressions of the digital companion. To successfully introduce a novel service, it is crucial to analyze users’ real-life behavior and adjust the service accordingly.

## 1. Introduction

Service design utilizes a human-centered approach focused on enhancing the user experience. Tangible and intangible elements experienced through the service are designed to provide a complete service experience for users. Understanding user intentions and behaviors can help ensure that specific needs are considered during service design. In the real-world environment where the service is experienced, service providers must aim to understand users and provide a centered total service experience. The value and quality of service experiences are improved by understanding the interaction between users and tangible and intangible service elements in a real-world environment. This becomes more important, particularly when developing a novel service that users have yet to experience. This is because by identifying life patterns in a real-life environment, necessary service design elements can be identified; moreover, repeated and immediate feedback from users can be observed in the living lab environment. The living lab environment enables the development of a design with enhanced usability and emotional aspects, overcoming the limitations of a fragmentary and one-sided product and service development process when considering user acceptability. The living lab is a concept in which user behaviors are analyzed, and services and products are investigated by applying science and technology in real-world environments for more realistic experiments [1,2]. This is a more active research method for improving the convenience of daily life and solving social problems. Living labs originated from the creation of Placelab, where users were observed using IT and sensor technology in a specific apartment that serves as a living space, at MIT Media Lab [2]. The narrow concept of a living lab, wherein users become the target of observation, has recently been expanded to a broader concept to allow citizens to actively participate in discovering urban problems and providing solutions. The concept of a living lab is widely used in these two aspects, with a specific focus on technologies related to key elements that are used by citizens and communities, such as housing, transportation, education, health, and energy.

The role of robots is becoming increasingly important as technology supports the daily lives of seniors, leading to studies on this topic. Service experiences have been designed based on prior studies that focus on understanding the issues related to the usability, safety, and sensibility of robots while considering the physical and cognitive aging as well as emotional characteristics of seniors [3,4,5,6,7,8]. In particular, digital companion robots that perform various roles, such as emotionally communicating with seniors and taking necessary measures in emergency cases, are anticipated as service robots that will become a key necessity in the future. By observing interactions in the real-world environments of seniors, a companion robot service can be designed to support natural interactions and functions in their daily lives. This will help achieve the ultimate goal of improving the quality of life among seniors and the general population.

This opinion piece aims to provide a comprehensive overview of living lab-based service interaction design through the introduction of a national R&D project, which serves as a case for developing a digital companion robot for seniors. This R&D project spanned approximately 3.5 years, during which seniors’ physical, emotional, cognitive, and language aspects were addressed, covering several factors ranging from functions to appearance. The project not only took into account these factors but also aimed to optimize the development of the robot for real-life usage in seniors’ living environments. It systematically established detailed objectives, set up suitable living lab environments for each goal, and devised evaluation methods for the robot, and this opinion piece shares the expertise gained from these endeavors. To present a viewpoint on utilizing a living lab and offer recommendations for future research in the field of developing robots, this opinion piece explores two perspectives: methodologies and considerations for the design of digital companion robot services. ‘Methodologies’ in this context refers to the various living lab methods that can be employed from the perspective of service interaction design for developing robots. It involves proposing methods applicable to different experiment objectives, providing details on how to utilize methods based on specific goals and how to configure the living lab environment, and suggesting the utility of these methods in different aspects. ‘Considerations’ outlines the essential factors and evaluation methods for robots targeting seniors, providing directions for future research in the field of service interaction design. It specifically focuses on the necessary considerations for interaction functions and the appearance of the robot. Furthermore, it presents the service design elements used as a basis for the evaluation and design of interaction functions and the robot’s appearance throughout the project. The primary contributions of this opinion pieceare as follows:

- It offers a perspective on the experimental environment and research directions for developing digital companion robots for seniors, which were not extensively considered in previous research. In particular, it can introduce elements to be considered when developing service design for seniors through actual experimental cases, providing practical guidance.
- It provides assistance in identifying research topics, ideas, and specific research methods in the relevant field. Taking into account seniors’ actual living environments and considering user experience, it introduces a multifaceted approach through various experimental settings, tools, and participants. This approach goes beyond isolated experiments and illustrates how service scenarios and prototypes for digital companion robots can be developed in diverse experimental contexts.

## 2. Materials and Methods

The emergence of the Fourth Industrial Revolution has led to attempts to apply new technologies, such as artificial intelligence (AI), to products and services. Users have yet to experience these services; thus, these services must be designed with the aim of improving the quality of life and convenience for users and should not focus solely on applying new technologies. In addition to improving convenience through new technologies, it is also essential to address social problems in the Fourth Industrial Revolution through service design. Technological innovation is becoming a key factor in solving various societal issues. The ultimate problem can only be solved by identifying user needs for future products and services rather than merely applying technology. Because users have no prior experience with the products and services they will use in the future, the role of service design is becoming increasingly important. Robots developed to address various social problems, such as the aging population, are novel products that most users have not encountered before. Usage patterns and behaviors must be identified and validated for newly created products and services, like robots. Therefore, it is necessary to develop the overall service experience and interaction for the robot by applying the service design method.

### 2.1. Living Lab

In particular, many studies are being conducted on service design based on living labs to address the social problems stemming from the increasing senior population in modern society. For instance, the CAPTAIN project in Europe serves as an experiment that provides smart assistance to help seniors lead independent lives and stay in their homes for as long as possible [9]. However, previous experiments involving seniors and new services have been limited because seniors often encountered difficulties when using complex or digital devices, resulting in ineffective outcomes. The CAPTAIN project successfully resolved these issues by leveraging the concept of living labs.

Transparent technology was proposed, taking into account the fact that seniors may not be familiar with new services. Microprojects involving projected augmented reality were implemented within a residential space, where experiments were conducted using a video interface. When seniors required assistance, a video was projected onto a nearby wall or table in their residential space to provide instructions. Once the assistance was no longer needed, the video automatically turned off, restoring the environment to a familiar residential setting.

The European Network of Living Labs (ENoLL) is a global open innovation ecosystem actively promoting living lab projects to develop user-centered products and services [10]. While living labs have been widely employed in various fields in Europe, their use in South Korea, although still limited, is gradually expanding. The case presented in this opinion piece marks South Korea’s first large-scale robot service design living lab project for seniors. While living lab methods generally provide diverse insights, there are limitations. Because the living lab method involves long-term observation and experimentation of daily life, recruiting participants can be relatively challenging. Living lab experiments necessitate a considerable amount of time, including the experimental and data analysis periods. The Active and Assisted Living (AAL) initiative in Europe aims to foster the development of products and services that truly impact people’s lives. This includes addressing the challenges of aging for individuals themselves and providing support for those who care for older people when assistance is required [11]. Among these, the ‘AgeWell’ project aimed to provide an avatar and robot-based personalized assistant to support the health and meaningful life of seniors. To achieve this, the project formed a user group involved in development from the early stages, and there were plans for long-term evaluations targeting this user group. The development incorporated the use of speech recognition technologies and machine learning, as well as scientifically proven methods and models from psychology.

### 2.2. Methods

**Figure 1:**
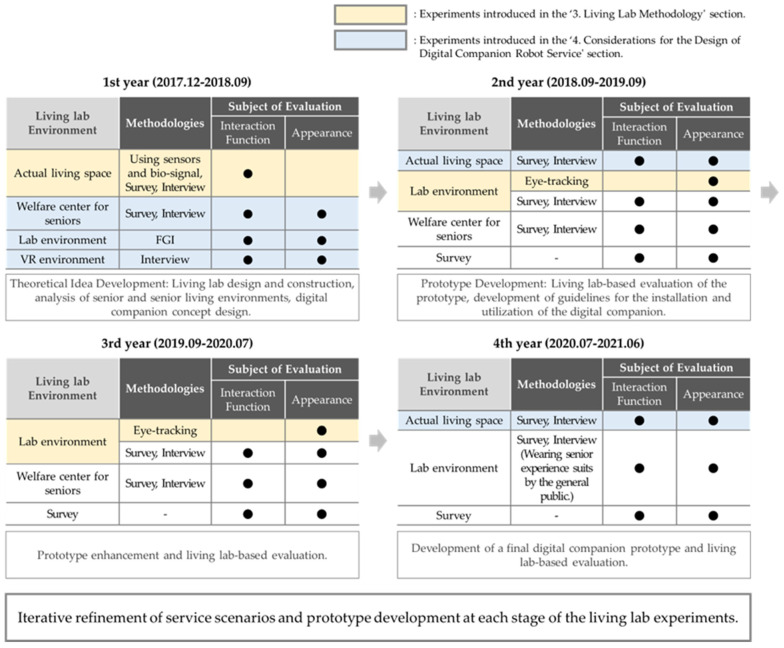
Overview of the living lab process in the R&D project.

This opinion piece provides methodologies and considerations for service interaction design within the living lab context, focusing on the development of a digital companion robot for seniors. In the context of the R&D project, the research questions revolve around determining the robot’s appearance and expression design; defining suitable interaction functions tailored to the physical, cognitive, and behavioral characteristics of seniors; and identifying the necessary service functions for seniors. We conducted a total of approximately 16 living lab experiments over a period of about three-and-a-half years (Figure 1). These experiments were broadly categorized into different living lab environments. Specifically, considering variations in evaluation subjects (such as seniors, senior-related experts, control groups, etc.), evaluation locations, specific evaluation methods, and criteria, we performed a greater number of experiments, establishing a highly diverse approach in experimental design. In the R&D project introduced in this opinion piece, numerous experiments were conducted. However, detailed methods and result analysis for all experiments are not provided. Instead, the focus is on presenting methodologies and considerations that can be helpful from a service interaction design perspective for future relevant research. We concentrate on introducing the step-by-step living lab process and highlighting the key insights derived from the experiments and methods in the R&D project. In the R&D project, a large-scale experiment involving 1491 participants was conducted, marking South Korea’s first large-scale robot service design living lab project for seniors (Table 1). This opinion piecewas carried out within a living lab environment, utilizing the living lab approach as a service design method. Life patterns and user characteristics were identified through experiments in real-life settings, with repeated evaluations and analyses of service design elements. The living lab environment included an actual living space, a laboratory space, a virtual reality (VR) environment, and a welfare center for seniors. Behavior analysis was conducted within the residential space using sensors, eye-tracking devices, interviews, and surveys. These methods facilitated the evaluation of usability, sensibility, acceptability, and safety-related factors, considering the functional, design, and interaction aspects of the service design elements defined in this opinion piece.

## 3. Living Lab Methodology

**Figure 2:**
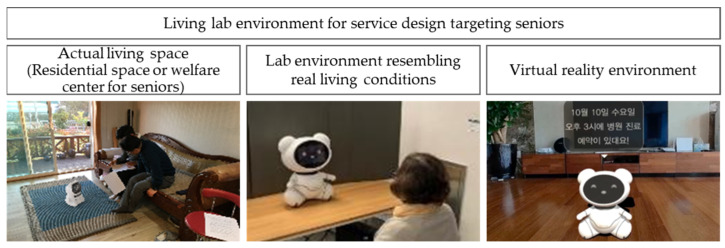
Living lab environment for service design targeting seniors.

Living lab environments include an actual living space, a lab environment resembling real living conditions, and a virtual reality environment [10,11,12,13,14,15]. In this opinion piece, we selected a living lab environment that aligns with the characteristics of the target users as well as the service functions and attributes (Figure 2). Depending on the focus of observation, various quantitative and qualitative methods, such as observation, surveys, interviews, and bio-signal measurements, were employed in the living lab environment to understand user needs.

In this section, we present selected cases from various living lab experiments of the R&D project to propose methods and factors to consider before prototype development for evaluating robots targeting seniors, beyond surveys and interviews. The living lab environment can be implemented not only for experimenting with finished products or prototypes but also with the sole purpose of analyzing the living environment and behavioral patterns of seniors. In this R&D project, we began the experimentation process by analyzing the living environment and behavioral patterns of seniors in an actual living space before prototype development, aiming to establish the foundation for robot development in seniors’ living environments.

For the experimental cases introduced in this opinion piece, we provide purposes for utilizing each method, advantages and limitations of the methods, and examples of the utilization direction of the experimental results.

### 3.1. Life and Behavior Patterns Using Sensors

Prior to capturing user needs, we examined the daily life patterns of the target users by installing sensors in the lab environment, extracting indoor activity data, and analyzing the observed behavioral patterns. We investigated the usage of daily life services by applying them within the living lab environment and installing sensors. A systematic understanding of service usability can be derived from the data on users’ living and behavioral patterns.

The cases presented in this opinion piece aimed to compare different age groups, encompassing individuals from their 30s to 70s. A total of 7 participants aged 30 to 40 and 12 participants aged 50 and above were recruited. The group of participants aged 50 and above comprised five males and seven females. Sensors were attached to the participants’ living spaces, and each experiment was conducted for two weeks in the year 2018. This experiment is identical to the one introduced in Section 3.2 and Section 3.3. The participants in each experiment were the same. The experiments specifically targeted single-person households and couples, excluding those living with family. This approach was chosen because the type of cohabitation can lead to differences in feelings of depression and life satisfaction. To address social issues, the experiments were conducted with the groups that showed relatively higher levels of depression and life satisfaction from the perspective of social problems.

**Figure 3:**
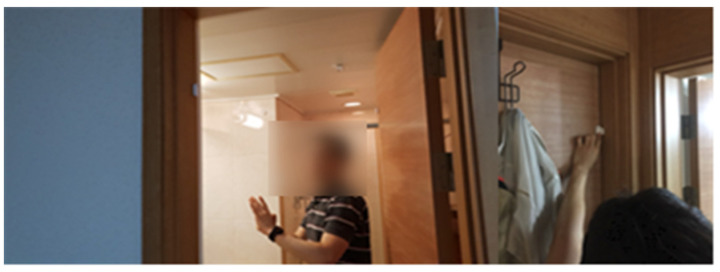
Installing sensors in the bathrooms and doors of the living spaces of senior participants.

**Figure 4:**
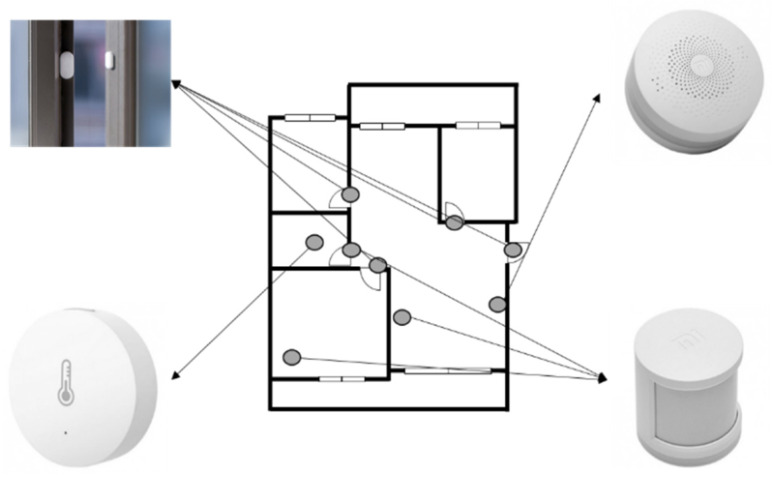
An example of sensor installation in a living space.

To develop a digital companion robot service for seniors, sensors were installed in the actual living environment to extract indoor activity data, including the frequency of space utilization, and to analyze living and behavioral patterns (Table 2). In addition to these quantitative data, a diary study method was employed, where senior users documented their daily routines. This combination of data sources enabled a systematic understanding of users’ living and behavioral patterns. The data derived from the diary study method complemented the limitations of the quantitative data collected with sensors. Living lab experiments involved multiple groups of individuals from various age groups to identify differences in their service experiences based on age. Motion, door, temperature, humidity sensors, and a wearable device were utilized to analyze the living and behavioral patterns of selected participants (Table 2, Figure 3 and Figure 4). The door sensor is a device attached to doors that detects real-time opening and closing movements. Considering factors such as the robot’s mobility, this sensor was used to comprehend spatial usage patterns and characteristics. The motion sensor employs infrared technology to detect objects and can perceive a maximum range of 170° horizontally and up to 7 m. This allowed us to determin whether seniors were present in specific areas. Taking into account the duration seniors spend in indoor spaces, the use of this sensor enabled the analysis of their daily living patterns. The temperature and humidity sensor identifies changes in temperature and humidity over time. This allowed us to understand seniors’ temperature regulation patterns and helped to determine the optimal temperature for their comfort. Moreover, in activities like cooking in the kitchen, the sensor can track actions through temperature and humidity variations. In addition, devices were installed for collecting data from these sensors. Using this information, the level of activity or time spent in each space was determined to design the service experience. Furthermore, these data can be leveraged to detect emergency situations and initiate an emergency call when there is no detected activity from seniors (Table 3).

### 3.2. Bio-Signal Measurement Using Wearable Smart Device

**Figure 5:**
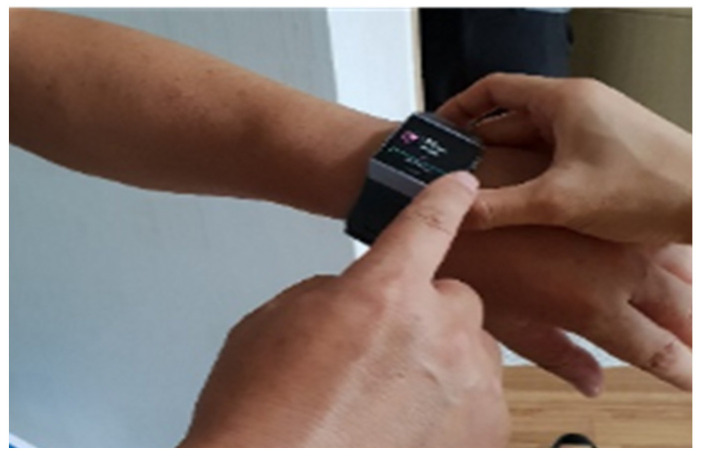
Bio-signal data collection using a smart device.

Measuring bio-signals using a wearable smart device was another method for analyzing living and behavioral patterns, in addition to installing sensors in the living space (Figure 4 and Figure 5). We used Fitbit’s smartwatch product, which measures steps and heart rate, in the living lab experiment. Activity and inactivity levels in daily life were analyzed using a smartwatch through step count. Moreover, we identified the correlation between activity levels and the tendency to use the digital companion robot and AI speaker by analyzing the data derived using this method. The activity level of senior participants, as determined using the smartwatch, varied depending on general characteristics such as age, gender, and sex. Male office workers in their 50s tended to show a high activity level with regular activities, such as going out for a run at 5 a.m. Female participants in their 60s tended to show a rapid decrease in physical activity levels compared to those in their 50s, and high activity levels were noted, especially early in the morning. The activity level decreases as people age. The functions of companion robots that seniors require can be determined based on their activity level. Moreover, the data can be used to determine whether there is a difference in the tendency to use digital companion robots depending on age, gender, and sex. This entails understanding the relationship between the usage patterns of AI speakers or prototype robots and the activity levels of seniors. For instance, when seniors exhibit low levels of activity, the derived data can be used to encourage features such as exercise or taking a stroll. Additionally, by examining the correlation between seniors’ feelings of depression and their activity levels, insights can be derived that help to brainstorm ideas for interaction functions aimed at addressing and alleviating depression (Table 4).

### 3.3. User Experience of AI Speaker

**Figure 6:**
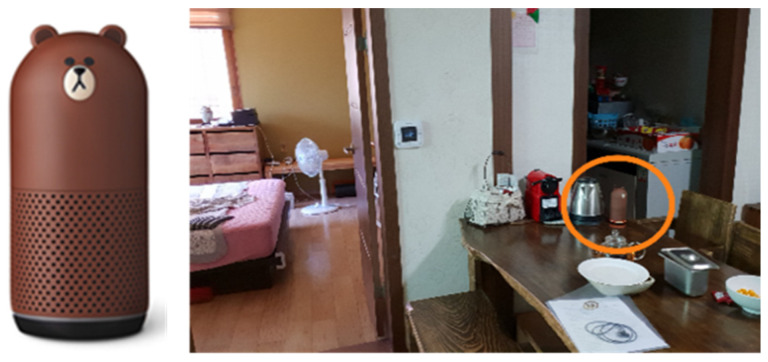
Installing the AI speaker Clova in actual living spaces.

An AI speaker can serve a similar role to a digital companion robot without mobility and can thus be installed to examine user familiarity with a digital companion, as well as the conversation patterns and necessary functions of the companion (Figure 6). Participants interacted with the AI speaker in their daily lives without any specific warnings or instructions to participate in the experiment. In this process, data on the linguistic behaviors and cognitive factors of senior participants were collected. Language characteristics were determined by conducting a living lab experiment in which emotional exchanges were possible when communicating with the companion robot, and such exchanges were detected through voice recognition. These characteristics can be used to develop technology that removes restrictions on voice recognition commands when using functions. The experiment was conducted in an actual residential space. The AI speaker used in this study was Clova, a voice-controlled speaker similar to Google’s Nest and Amazon’s Echo [16].

Clova engaged in daily conversations with users by recognizing their voices and provided a range of services, including information retrieval, shopping, music playback, conversation, alarm setting, and weather checking [16]. Findings from the living lab experiment indicate that seniors most frequently used functions related to music, lifestyle information, and conversation. A notable finding from the experiment was that almost half of the total artificial intelligence (AI) speaker usage of seniors involved listening to music [16]. According to this study, seniors showed a significantly higher preference for music than younger age groups.

For seniors living alone, the AI speaker Clova served as a companion with whom they emotionally interacted. Seniors conversed with the AI speaker in a manner resembling conversations with a human friend. They made comments like “It’s been a while”, “I missed you”, “Good night”, “I was a little sad”, and “How can I express my feelings toward someone I love”? Remarkably, senior users even confided their personal problems to the AI speaker. Over the course of the 14-day household experiment, a total of 1600 voice commands were collected and analyzed remotely [17]. The total number of interactions observed among seniors exceeded that of the younger age group, largely due to seniors spending more time at home. Notably, the total number of failed function attempts while using the AI speaker did not significantly differ between seniors and their younger counterparts. The information obtained through this experiment and the subsequent data analysis were crucial in identifying essential functionalities for developing robots tailored to seniors’ needs. Not only did the analysis of voice command data reveal seniors’ preferred functions, but it also allowed for an understanding of the linguistic nuances in the emotional interaction when seniors engaged with the robot (Table 5).

**Figure 7:**
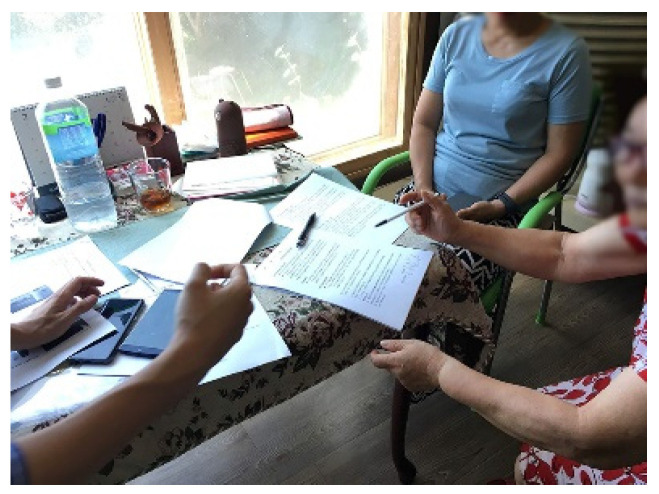
Conducting the ex-post survey and interview.

**Figure 8:**
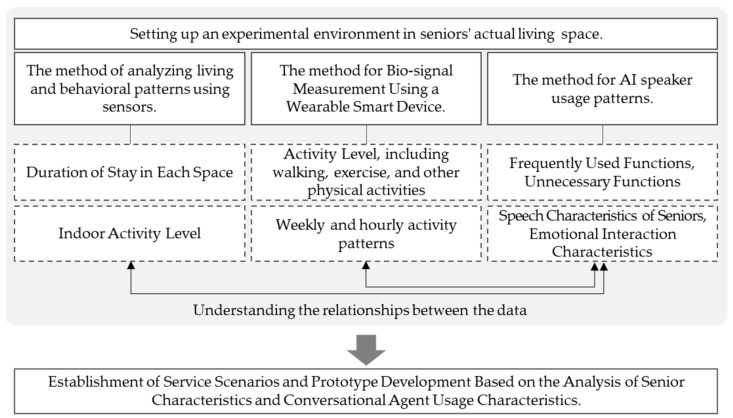
Utilization of living lab experiment results.

After the living lab experiment ended, an ex-post survey and interviews were conducted to ascertain the detailed requirements of the participants (Figure 7). The interviews were aimed at collecting qualitative data and subjective user opinions to precisely analyze the required service functions, in addition to the data gathered from residents’ daily lives. Figure 8 provides an overview of the living lab experiment introduced above, outlining the possibilities for data utilization.

### 3.4. Eye-Tracking Experiments and Appearance

**Figure 9:**
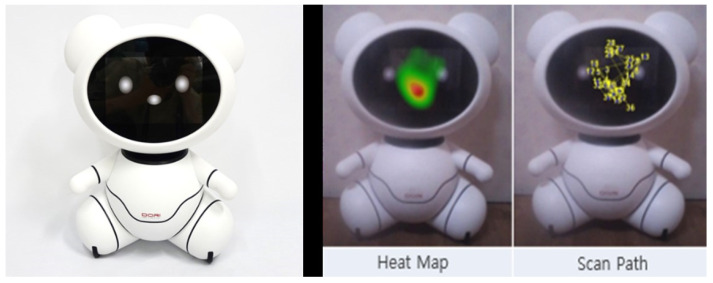
Observing the use of the prototype with an eye-tracker device.

**Figure 10:**
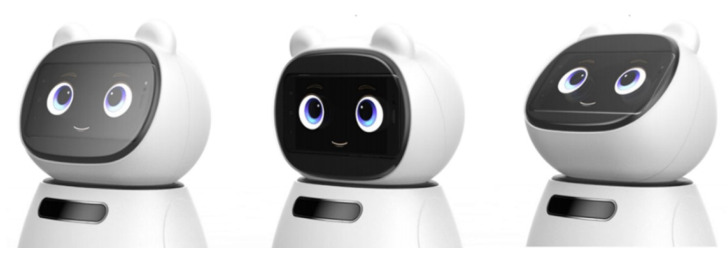
An example of a companion robot developed with high acceptability based on the results of the living lab experiment.

The eye-tracking experiments in the R&D project were conducted twice. The first experiment was performed in March 2019, collecting and analyzing data from 31 individuals aged 50 and above, and a control group of 31 young adults aged 18 to 29. The second experiment was conducted from November to December 2018, involving 10 individuals in their 60s and a control group of 10 individuals in their 20s. As the living lab experiments progressed in stages, the prototype’s appearance and interaction functions were continuously modified and developed. Therefore, the prototype used in the first eye-tracking experiment with the utilization of eye trackers differed from the one used in the second experiment. However, both robots used in the two experiments were teddy bear-like robots. In both the first and second experiments, the senior group tended to gaze at the robot’s facial area for a longer duration than the control group. It was observed that the senior group, compared to the control group, showed a more prolonged gaze at the robot’s facial area, concentrating their attention on the facial region rather than other areas of the robot (Table 6, Figure 9) [8]. Based on the experiments conducted prior to the eye-tracking experiment for prototype development, seniors showed a preference for teddy bear-like robots. Therefore, in this R&D project, the prototype was evolved based on the teddy bear form. Additionally, during the eye-tracking experiment, participants evaluated the appearance of the robot. It was determined that, compared with younger individuals, seniors favored teddy bear-like robots. Therefore, in the R&D project, a teddy bear-shaped robot was designed, allowing for neck rotation in all directions (Figure 10).

## 4. Considerations for the Design of Digital Companion Robot Service

**Figure 11:**
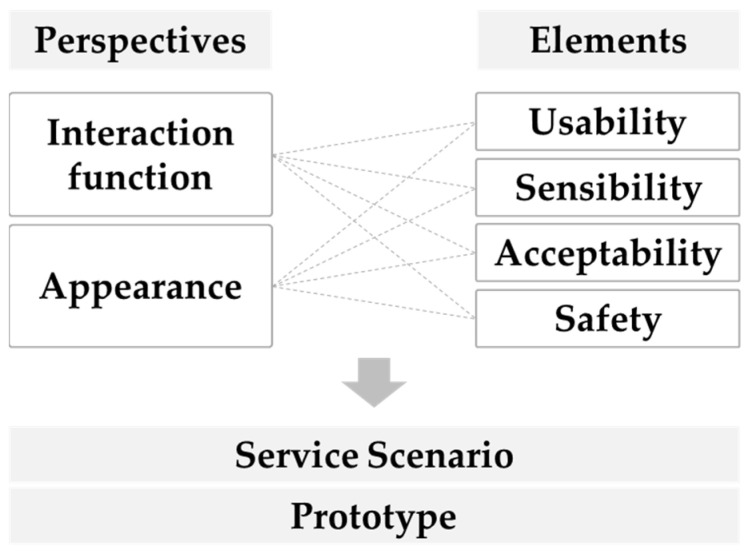
The overarching concept for the design considerations of a digital companion robot service.

During the R&D project, we aimed to identify crucial factors from the perspectives of interaction function and appearance (Figure 11). We carried out a validation procedure for the service scenarios and prototype, focusing on usability, sensibility, acceptability, and safety aspects. This section introduces cases of living lab experiments conducted in the R&D project.

In the R&D project process, the tools and scales used in each experiment utilizing the prototype were structured to encompass both interaction function and appearance aspects. Excluding survey questions that focused on general characteristics and specific scales, most questions were conducted using a Likert five-point scale. Generally, participants were asked to respond to topics such as service satisfaction, usability, product intimacy, satisfaction with appearance, satisfaction with impression, sensibility and emotion, system usefulness, and safety. In addition to questions related to the characteristics of robot use, data on general and emotional characteristics (e.g., sex, age, household type, personal income, depression, etc.) were also collected.

### 4.1. Service Scenarios and Prototype Development

#### 4.1.1. Definition of Requirement-Based Service Scenarios

**Figure 12:**
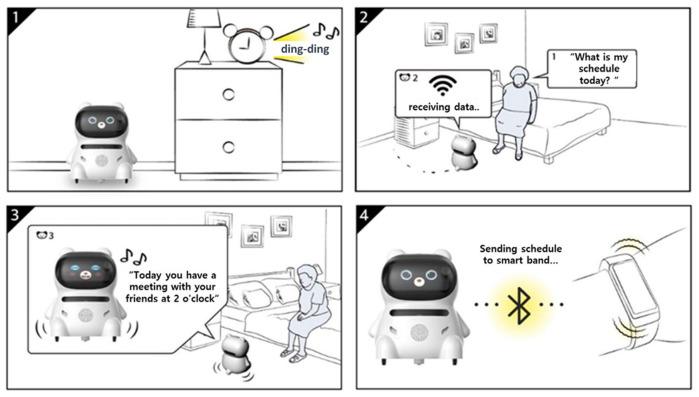
An example of a service scenario.

Service scenarios were developed based on the requirements derived from the R&D project’s living lab experiments, followed by the development of a digital companion robot prototype. Scenarios were developed and validated through living lab experiments to meet the requirements of seniors. Based on the analysis of senior characteristics in the first year of the R&D project and the analysis of living lab experiments, we developed 70 scenarios. Through incremental adjustments and improvements during the staged progress of the living lab experiments, a total of 88 scenarios were finalized in the third year. The developed scenarios included ‘scheduling and weather-related scenarios’, ‘entertainment-related scenarios’, ‘household-management-related scenarios’, ‘technology and internet-use-related scenarios’, ‘health-related scenarios’, ‘communication-related scenarios’, etc. Figure 12 illustrates an example scenario for notifying a schedule.

#### 4.1.2. Prototype Development

Since the details about interaction functions are covered in Section 4.2.2, this section introduces the factors considered in terms of appearance during prototype development. The content presented here is based on the analysis of results from living lab experiments conducted as part of the R&D project until the 4th year. Instead of focusing on experimental outcomes, this section outlines the elements that were taken into account during the development process.

**Figure 13:**
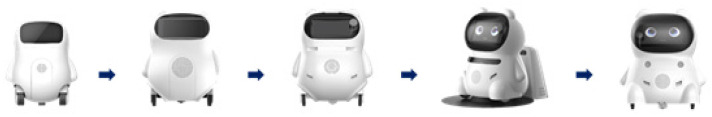
An example of concept design development with high acceptance.

Several aspects were revealed through living lab experiments involving prototypes. These experiments assessed the companion robot’s usability and user preferences. Design requirements related to the robot’s appearance, auditory feedback tailored to seniors’ hearing abilities, and the tactile properties of materials were taken into account. The size of the robot was also considered, and opinions favoring its compact size for emotional reasons were noted. Negative opinions about the robot’s size were rarely expressed. Additionally, inquiries were made about the overall form of the robot, as well as the size of the display screen and the perception of images and text displayed on the screen. Satisfaction with these aspects was met in the final prototype. While the size of the robot, considering the sedentary lifestyle of seniors, was deemed important, there was a consistent preference for the robot to be lightweight for easy carrying, aligning with the physical characteristics of seniors. Some seniors even expressed a tendency to pick up and embrace the robot during use. Safety perceptions, including concerns about tripping or instability, were also investigated. Overall, the sense of safety was reported to be at least ‘average’, and the aspect of safety related to the robot’s structure showed a relatively high level of confidence, with respondents indicating that there was no significant risk of injury due to the robot’s design. It was observed that seniors favored a teddy bear-like design for the robot. The robot’s appearance also encompassed the design of facial expressions. Seniors expressed the need to easily discern situations or the robot’s emotions through its facial expressions. Consequently, facial expressions were developed to convey a range of emotions, including neutrality, happiness, surprise, fear, anger, and sadness. By conducting living lab experiments, we proposed a robot concept that is well suited to the living environment of seniors (Figure 13).

### 4.2. Interaction Functions and Appearance

#### 4.2.1. Service Requirements

During the R&D project period, continuous living lab experiments were conducted to develop and refine service scenarios and prototypes and to establish a digital companion development strategy [6,7,8,17,18,19]. Among these experiments, visits were made to actual residential environments and welfare centers for seniors to gather and analyze service requirements from the perspective of seniors. This included their psychological characteristics, lifestyle features, perceptions of the digital companion’s appearance (material, form, and type), satisfaction with interaction functions, intimacy, and overall impressions. Additionally, focus group interviews were conducted with professionals working with seniors, such as social workers and caregivers, to collect requirements related to seniors’ cognitive behavior, as well as their preferences for the appearance and functions of the digital companion. The reason for conducting focus group interviews with experts related to seniors was to understand the cognitive and behavioral characteristics of seniors, define functions through identifying cases such as the types and frequency of emergency situations, and develop scenarios. This was conducted in April 2018 with a total of nine social workers, caregivers, and nurses. The interviews were conducted in groups of four and five, with each group lasting for two hours.

The service requirements based on the interviews with professionals working with seniors are presented in Table 7.

#### 4.2.2. Preferences for Appearance and Functions Based on Scenario Using Virtual Reality

**Figure 14:**
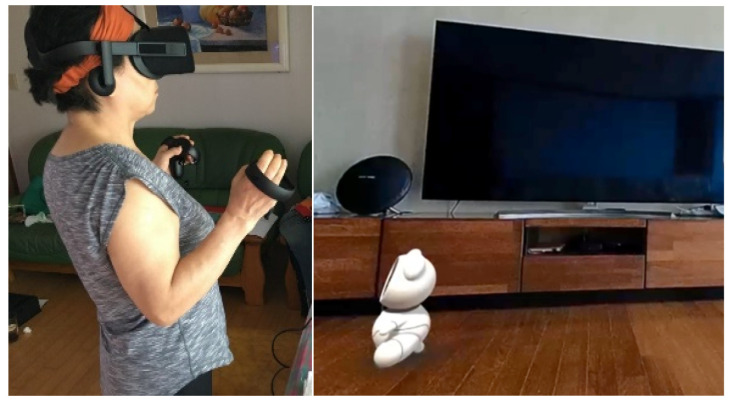
Virtual reality experiment.

We captured the various spaces of the actual home environments of seniors and constructed a virtual living lab environment. Additionally, we built a conceptual model of a digital companion and used it in the virtual space. We recreated the actual living spaces of seniors, including bedrooms, living rooms, kitchens, etc., in virtual reality. The experiments were conducted with a total of 57 seniors from December 2018 to January 2019. This allowed us to conduct validation on the robot’s appearance and functions according to different usage scenarios for seniors (Figure 14). The results indicated that among the various appearance types, seniors expressed a preference for digital companions in the form of animal-like characters. Furthermore, it was found that providing a proactive conversation function, where the digital companion initiates dialogue with seniors, was more suitable than delivering specific functional features within the scenarios. Through virtual reality experiments, it became evident that the experiment duration and costs were significantly reduced compared to the experiments conducted in a real-life living lab environment.

#### 4.2.3. Interaction Functions

**Figure 15:**
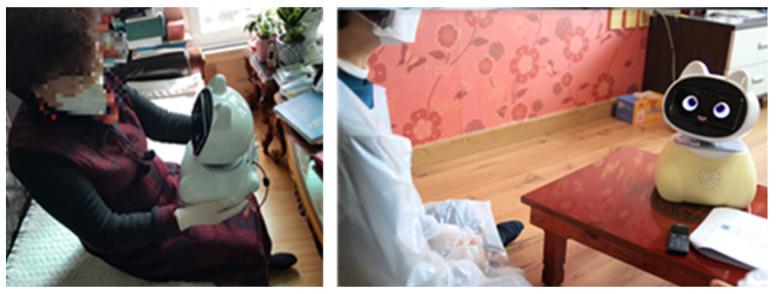
Living lab experiments on interaction functions.

In the living lab environment, we conducted experiments to evaluate satisfaction with interaction functions, not only utilizing AI speakers but also developing prototypes (Figure 15). Experiments with the prototype were conducted twice in seniors’ actual living spaces. The first experiment was performed in August 2019 with 16 seniors (7 males and 9 females), and the second experiment was conducted in April 2021 with 8 seniors (3 males and 5 females). Table 8 provides an example list of functions that seniors experienced during the experiment. Among the various service functions, the ‘general calling feature’, ‘alarm setting feature’, ‘low battery notification feature’, ‘temperature measurement feature’, ‘volume control feature’, ‘schedule reminder feature’, and ‘emergency call feature’ exhibited relatively high satisfaction. The ‘conversation feature’ was consistently included in seniors’ demands, but it showed relatively low satisfaction due to the prototype robot’s responses not being sufficiently fast.

## 5. Discussion

Studies have been conducted on user experience and the interaction between robots and users from a service design perspective [20,21,22,23,24,25,26,27,28,29,30,31,32,33,34,35,36,37,38,39,40,41]. The R&D project introduced in this opinion piece focused on using the living lab approach as a service design method to enhance the overall service experience.

The content presented in this section highlights the crucial elements derived from the experimental results of the R&D project. While it includes information about the experimental results, it also illustrates considerations from a user experience perspective for the development of a single robot. Additionally, it emphasizes the versatility of analysis when using the living lab method.

The group with a high level of depression preferred the bear type, whereas the group with the lowest level of depression favored the newborn baby type [19]. It was observed that seniors’ preference for newborn babies was rooted in their desire to see their grandchildren, and it was found that the preference for the bear type was associated with a need for emotional stability. Regarding physical characteristics, it was noted that the robot tended to feel heavy when its weight exceeded 1 kg [19].

The recurrent and immediate feedback from seniors can be effectively incorporated into living labs, thereby addressing the limitations associated with fragmentary and one-sided acceptance. This process aids in the development of a design characterized by improved form, aesthetics, and sensibility. A crucial element involves the implementation of accessible and natural interactions, with a focus on usability, and the inclusion of interactions that ensure user-friendly experiences. The concept of ‘interactions that guarantee ease of use’ revolves around minimizing the user’s time and effort. Taking into account this facet of usability and tailoring the robot’s movements and forms to suit seniors’ living environments, companion robot concepts and services can be crafted to enhance their acceptability among seniors. By understanding seniors’ emotional preferences, we were able to propose a concept design for a companion robot in the form of a teddy bear, which could foster a sense of familiarity among seniors, particularly those unfamiliar with robots. Furthermore, the curved shape of the robot provided comfort to seniors, and they expressed a preference for a simplified antenna. The residential spaces of seniors in South Korea should be reconsidered to reduce movement restrictions, considering the numerous obstacles to robot mobility that are attributed to the sedentary lifestyles of seniors.

It is necessary to investigate to what extent the continuous and prolonged use of robots from an ethical standpoint genuinely benefits seniors. Although the experimental results with seniors targeted a positive aspect of the robot’s service design, it does not necessarily imply that the use of robots positively impacts seniors’ overall lives. Understanding the level of influence and whether the extended use of robots is indeed beneficial for seniors in practical terms becomes crucial. While the experiments with seniors yielded positive results concerning the service design of the robot, it does not necessarily imply that the use of robots has a uniformly positive impact on overall life. Negative effects or ethical concerns related to utilizing robots to address social issues should be addressed in future research, alongside considerations of service design aspects.

### 5.1. Living Lab Methodologies

A digital companion robot was designed for an R&D project with the goal of improving the acceptance and quality of life for seniors. As for the research methodology, a living lab was established to collect data on the cognitive, emotional, and behavioral characteristics of seniors, enabling repeated evaluations and analyses. Additionally, a network was built to examine the living environment of seniors in collaboration with the local community.

Living lab experiments must be comprehensively designed to find service design elements suitable for the dynamic contexts of users. To this end, the living lab experiment in this opinion piecewas conducted in actual living spaces or at welfare centers visited by seniors. Eye-tracking and virtual reality (VR) experiments were also conducted to determine the service requirements and further develop the appearance of the robot. Moreover, in-depth interviews were conducted with individuals involved with seniors to understand their physical, cognitive, verbal, and behavioral characteristics. When conducting experiments with prototypes, validation methods can vary depending on the living lab environment (e.g., actual living space, lab environment, etc.). Even in cases with repeated validation in the same environment, experiments can be conducted with different tools or methods based on the service design elements to be evaluated. Additionally, within the same living lab experiment, control groups can be included as experiment participants to analyze the differences with seniors. Furthermore, diversifying the participants, including experts related to seniors, allows for the derivation of a service interaction design optimized for the living environment of seniors. Repeated and continuous living lab experiments proved effective in designing companion robot services that can enhance the quality of life for seniors.

### 5.2. Service Design Elements

When designing a digital companion robot service, it is essential to consider service design elements from the perspectives of usability, sensibility, acceptability, and safety. Consequently, design development was guided by a combination of these service design elements, based on meaningful results derived from the living lab experiment (Table 9). Table 9 provides a comprehensive summary of service design elements identified as useful for evaluating the use of robots among seniors, based on the design details of all living lab experiments conducted in the R&D project introduced in thisopinion piece. For instance, regarding the satisfaction elements associated with robot impressions, notable differences and requirements were observed among seniors when comparing fixed facial expressions with changing expressions, such as when the robot blinked its eyes. Usability is a factor associated with the design of practical and dependable services that seniors can easily utilize. In contrast, acceptability is associated with crafting services that align with user behavior and cater to the physical and cognitive characteristics of seniors. Sensibility pertains to how much trust, safety, and satisfaction a user experiences when interacting with a robot. The safety factor is concerned with the robot’s mobility, potential collisions, product durability, and the risk of fire occurrence.

There were instances of slow robot responses, voice recognition responses, errors in conversation, and issues with the robot’s gaze tracking function, which led to low satisfaction regarding technical aspects. This is based on the data derived from the experiments introduced in Section 4.2.3 and the analysis of negative experiences during the use of the prototype, which were obtained through interviews in which participants were questioned about their experiences. However, beyond these technical aspects, the prototype developed in each stage of the R&D project was continuously optimized for service design that aligns with the characteristics of seniors, and overall, seniors expressed a high preference for the digital companion robot. Some aspects that were found to be significant for the prototype’s design are highlighted below, providing insights for future research.

Adequate screen size and font size are considered key factors in usability. In terms of appearance, there is a need for a design that reduces resistance and increases familiarity with the robot, as seniors are typically unfamiliar with robots. Concerning the impression, the mechanical aspect must be minimized by ensuring the robot’s facial expressions are more direct and gentle, presenting a natural and trustworthy impression.

According to the findings, there was a high dependency on and expectations for the emergency call function in case of emergencies; therefore, a high-level voice recognition technology should be provided. It is necessary to understand the language characteristics of seniors. Among seniors, the diversity of words used was low, and fluency decreased. It was also observed that certain voices and tones were preferred by older individuals, such as those of young women. Regarding usability, satisfaction with using content by touching the display attached to the robot was found to be high. However, some stated that it was difficult to touch the screen due to the small size of the display. In terms of satisfaction with the appearance, factors such as attractiveness, intimacy, and aesthetics of the robot were identified. A negative opinion was expressed that the plastic material appeared rigid. If the robot’s material or movements feel mechanical and lack a sense of vitality, it could be challenging to establish intimacy. Concerning satisfaction with impressions, seniors expressed a high level of satisfaction with the robot’s reactions to the user through its eyes; the robot’s eyes were considered the factor that made it feel more like an organic life form. If the robot’s gaze processing and focus become more apparent during interactions, the mechanical sensation can be reduced.

## 6. Conclusions

The R&D project introduced in this opinion piece is a large-scale living lab research study conducted in South Korea targeting seniors. The study advanced to the development of a highly refined prototype, and as a result, in terms of practical experiential aspects, the following conclusions were drawn: A living lab can be utilized to design both tangible and intangible elements, creating a comprehensive service experience for seniors using digital companion robots. A human-centered, holistic experience becomes achievable by understanding the interaction between users and both tangible and intangible service elements in a real-world environment. For a novel service that seniors may not have prior experience with, it is essential to observe user behavior in a real-world environment and analyze living and behavioral patterns.

A living lab environment encompasses an actual living environment, a laboratory setting resembling a real living environment, and a virtual reality environment.

To conduct a precise analysis of the required functions, a method must be employed to collect subjective user opinions, such as through post-experiment surveys, in addition to the data naturally acquired from seniors in their daily lives. From a service design perspective, even if interaction functions or appearance aspects are well considered and designed, satisfaction may be low if the robot does not respond quickly in terms of the technical aspect of interaction with seniors. Therefore, technical aspects should also be taken into account to ensure satisfaction, besides well-designed interaction functions and appearance from the service design standpoint. To ensure that service design elements are comprehensive, various living lab environments must be established, and methods must be thoughtfully designed to discern user needs. Through the implementation of repeated and continuous living lab experiments, services can be designed to enhance usability, sensibility, acceptability, and safety.

## Figures and Tables

**Table 1:** Living lab experiment with 1491 participants.

Year of the Experiment	Living Lab Environment	Total Number of Participants in Each Experiment
20 December 2017–19 September 2018	Actual living space/Lab environment/ Welfare center for seniors/VR environment	320
20 September 2018–19 September 2019	Actual living space/Lab environment/ Welfare center for seniors/Survey	700
20 September 2019–19 July 2020	Lab environment/Welfare center for seniors/Survey	56
20 July 2020–19 June 2021	Actual living space/Lab environment/ Survey	415

**Table 2:** Types of sensors used for life and behavior pattern analysis.

Type of Sensor	Function
Door sensor	Senses the door opening and closing in real time
Motion sensor	Senses objects at a certain distance by using infrared rays
Temperature and humidity sensor	Senses changes in temperature and humidity on an hourly basis
Wearable device	Senses bio-status and locations in real time

**Table 3:** The method of analyzing living and behavioral patterns using sensors.

Purposes for Utilizing the Method	Advantages and Limitations of the Methods	Examples of Utilization Direction of the Experimental Results
The purpose is a multifaceted analysis of the living and behavioral patterns of specific targets such as solitary seniors and couples.	Objective and quantitative data collection is possible, providing a foundation and validity for the development of robot functions and services, thereby reducing errors in prototype development. Due to the nature of the experiment, which requires considering participants’ living spaces, participant recruitment is challenging, and the overall measurement of daily life takes longer than typical experiments.	Interaction functions are considered based on activity levels and the time spent in each space. The utility of the digital companion robot is assessed based on the extent and patterns of movement indoors, taking into account situations where the robot moves on the floor or seniors carry the robot, ensuring that the exterior design is not limited.

**Table 4:** The method for bio-signal measurement using a wearable smart device.

Purposes for Utilizing the Method	Advantages and Limitations of the Methods	Examples of Utilization Direction of the Experimental Results
The analysis of seniors’ activity levels aims to identify gender- and age-related differences and extract suitable functionalities accordingly.	Since the experiment conductor does not directly observe the participants’ behavior in real time, supplementary methods such as diary studies are necessary to enhance the accuracy of the data. Many seniors may take time to become accustomed to using wearable smart devices, especially when using them for the first time.	The activity levels of seniors vary based on individual factors, allowing for the selection of necessary features for each target group. Activity levels may be correlated with factors such as depression, and through the analysis of the relationship between seniors’ cognitive, physical, emotional, and linguistic aspects, among other elements that are crucial for robot development, ideas for functionalities that assist seniors in their daily lives can be derived.

**Table 5:** The method for AI speaker usage patterns.

Purposes for Utilizing the Method	Advantages and Limitations of the Methods	Examples of Utilization Direction of the Experimental Results
The goal is to analyze the usage patterns of AI speakers, focusing on the functions seniors frequently use and analyzing conversational features, including emotional interactions.	When recruiting participants for the experiment, considerations for creating the experimental environment are necessary, especially since there are cases where Wi-Fi may not be installed in seniors’ living spaces. Therefore, when analyzing interaction characteristics using devices such as AI speakers, it is important to account for variations in the availability of Wi-Fi within seniors’ residences.	In addition to analyzing frequently used functions and unnecessary functions through AI speaker usage data, it is possible to identify potential functionalities based on what seniors express to the AI speaker, even if those functionalities are not currently integrated into the device. Analyzing language data reveals that seniors tend to perceive AI speakers as conversation partners, and understanding the prevalent emotional interactions can provide insights for shaping the feedback and conversational characteristics of future digital companion robots.

**Table 6:** The method for AI speaker usage patterns.

Purposes for Utilizing the Method	Advantages and Limitations of the Methods	Examples of Utilization Direction of the Experimental Results
The goal is to use eye-tracking devices to analyze gaze factors related to the robot for the design of the robot’s appearance.	The data collected for appearance include both subjective data gathered through surveys and interviews with experimental participants and objective data. Through comprehensive analysis of the data, important factors related to appearance can be derived. Since participants needed to maintain a fixed posture during the experiment, their movements were restricted, and there was occasionally a risk of failure during the calibration process after wearing the eye-tracker device. Therefore, there is also a possibility that data might not have been collected for some of the recruited participants.	Seniors tend to focus their gaze on the facial area rather than scanning the overall form of the robot broadly. This tendency highlights the importance of morphological factors such as facial shape and expressions.

**Table 7:** The companion robot service requirements for seniors.

Service Requirements
Ability to help seniors locate frequently misplaced items.
Assistive function for medication management among seniors.
Hospital reservation feature.
Notification function for identifying potential risks in the vicinity of seniors.
Life pattern analysis and emergency response capabilities.
Proactive health status checks and telemedicine features for seniors through the digital companion.
Dining conversation feature/nutrition management capability.
Welcoming and waiting for seniors at the door.
Reminders for new programs and schedules at senior centers.
Recommendations for walking and physical activity.
Displaying photos of grandchildren based on seniors’ preferences.
Ability to use the digital companion’s voice as that of a grandchild.
Game content for seniors aimed at cognitive rehabilitation and emotional well-being.

**Table 8:** Interaction function examples.

Function	Voice Command
Alarm	Set the alarm.
Call	General call—show me the list of calls.
	Emergency call—show me the emergency phone number list.
Music	Play music.
Photo	Show photos.
Diary	Let’s write in my diary.
Game	Let’s play a game.
Video	Show me a video.
Menu	Show me the menu.
Help	What can I do?
Volume control	Turn up/turn down the volume.
Timer for medication	Set the timer for medication.
Temperature measurement	Measure my temperature.
Preemptive measures	Request conversation when the user does not speak./Make an emergency call when an abnormality is detected in the user./Notify the user about the schedule set in advance.

**Table 9:** Service design elements for developing digital companion robots.

Category	Content
Service satisfaction	Satisfaction with the functions currently provided by the robot.
Usability	Display UI/GUI, response time, operation mode, voice and tone (language), overall convenience, etc.
Product intimacy	Formation of companionship, satisfaction with conversations, emotional connection with the robot, etc.
Satisfaction with appearance	Satisfaction with the robot’s mobility, familiarity, materials, etc.
Satisfaction with impression	Satisfaction with the facial expressions on the display, the naturalness of the expressions, and overall impression (kind, wise, dynamic, lively, etc.).
Sensibility and emotion	Trust, stability, pleasure, etc.
System usefulness	Usage, learnability, pride, robot’s stability, etc.
Safety	Risk of electric shock, maintaining stability without shaking, etc.

## Data Availability

Data are contained within the article.
